# A Novel Individual Metabolic Brain Network for 18F-FDG PET Imaging

**DOI:** 10.3389/fnins.2020.00344

**Published:** 2020-05-12

**Authors:** Sheng-Yao Huang, Jung-Lung Hsu, Kun-Ju Lin, Ing-Tsung Hsiao

**Affiliations:** ^1^Department of Medical Imaging and Radiological Sciences, Healthy Aging Research Center, Taoyuan, Taiwan; ^2^Molecular Medicine Research Center, Chang Gung University, Taoyuan, Taiwan; ^3^Department of Neurology, New Taipei Municipal TuCheng Hospital, New Taipei City, Taiwan; ^4^Department of Neurology, Chang Gung Memorial Hospital Linkou Medical Center and College of Medicine, Neuroscience Research Center, Chang-Gung University, Taoyuan, Taiwan; ^5^Graduate Institute of Humanities in Medicine and Research Center for Brain and Consciousness, Shuang Ho Hospital, Taipei Medical University, Taipei, Taiwan; ^6^Department of Nuclear Medicine and Molecular Imaging Center, Linkou Chang Gung Memorial Hospital, Taoyuan, Taiwan

**Keywords:** individual metabolic network, FDG-PET, Alzheimer’s disease, progressive MCI, stable MCI

## Abstract

**Introduction:**

Metabolic brain network analysis based on graph theory using FDG PET imaging is potentially useful for investigating brain activity alternation due to metabolism changes in different stages of Alzheimer’s disease (AD). Most studies on metabolic network construction have been based on group data. Here a novel approach in building an individual metabolic network was proposed to investigate individual metabolic network abnormalities.

**Method:**

First, a weighting matrix was calculated based on the interregional effect size difference of mean uptake between a single subject and average normal controls (NCs). Then the weighting matrix for a single subject was multiplied by a group-based connectivity matrix from an NC cohort. To study the performance of the proposed individual metabolic network, inter- and intra-hemispheric connectivity patterns in the groups of NC, sMCI (stable mild cognitive impairment), pMCI (progressive mild cognitive impairment), and AD using the proposed individual metabolic network were constructed and compared with those from the group-based results. The network parameters of global efficiency and clustering coefficient and the network density score (NDS) in the default-mode network (DMN) of generated individual metabolic networks were estimated and compared among the disease groups in AD.

**Results:**

Our results show that the intra- and inter-hemispheric connectivity patterns estimated from our individual metabolic network are similar to those from the group-based method. In particular, the key patterns of occipital-parietal and occipital-temporal inter-regional connectivity deficits detected in the groupwise network study for differentiating different disease groups in AD were also found in the individual network. A reduction trend was observed for network parameters of global efficiency and clustering coefficient, and also for the NDS from NC, sMCI, pMCI, and AD. There was no significant difference between NC and sMCI for all network parameters.

**Conclusion:**

We proposed a novel method in constructing the individual metabolic network using a single-subject FDG PET image and a group-based NC connectivity matrix. The result has shown the effectiveness and feasibility of the proposed individual metabolic network in differentiating disease groups in AD. Future studies should include investigation of inter-individual variability and the correlation of individual network features to disease severities and clinical performance.

## Introduction

Alzheimer’s disease (AD) is a major neurodegenerative disease with clinical characteristics of memory and cognitive decline due to structural and functional changes in the brain. Early detection of AD, even before the transitional stage of mild cognitive impairment (MCI), is necessary and important for potential disease prevention and treatment. To capture the structural and functional changes in the brain due to disease, various tracers in positron emission tomography (PET) were investigated for imaging neuropathological changes using amyloid and tau PET imaging (Iturria-Medina et al., 2014; Oxtoby et al., 2017; Hanseeuw et al., 2019) and for imaging metabolism (Kuang et al., 2019).

In addition to the imaging quantitation, brain network analysis based on graph theory using neuroimaging methods provides network information about brain organization and has recently become a potentially useful diagnostic tool for investigating functional or structural connectivity changes in neurodegeneration (Raj et al., 2015; Garbarino and Lorenzi, 2019; Garbarino et al., 2019). In particular, metabolic network analysis using FDG PET provides functional interregional connectivity information and has been reported to offer differential diagnosis power for different disease groups in AD (Seeley et al., 2009; Sanz-Arigita et al., 2010; Huang et al., 2018). However, unlike fMRI (Zhou et al., 2012), which includes time-series information, metabolic networks derived from static FDG PET scans are usually constructed from group data (He et al., 2008; Seo et al., 2013; Duan et al., 2017; Huang et al., 2018), and only group-level network properties can be studied (Kuang et al., 2019). However, to investigate individual brain abnormalities and inter-subject variability, an individual brain network is necessary for single subjects. To solve this problem, a few methods for deriving individual brain networks have been proposed and most of them involved morphological network construction using T1-weighted MR images based on regional distance measurements (Raj et al., 2010; Zhou et al., 2011; Tijms et al., 2012; Kong et al., 2015; Li et al., 2017), regional morphological distributions (Kong et al., 2015), network diffusion models (Raj et al., 2012), or multi-voxel nodes (Tijms et al., 2012). Only a few approaches for constructing individual metabolic networks have been proposed recently based on multi-voxel cubes (Yao et al., 2016), multimodal connectivity (Iturria-Medina et al., 2018), or regional intensity relations (Li et al., 2018).

In this study, we proposed a novel approach for constructing individual metabolic networks using a static FDG PET image for single subjects. The method is based on calculating a weighting matrix from the interregional effect size (ES) difference between a single subject and average normal controls (NCs), and then imposing the weighting matrix on a group-based connectivity matrix of a NC cohort. To study the performance of the proposed method, inter- and intra-hemispheric connectivity patterns in groups of NC, stable mild cognitive impairment (sMCI), progressive mild cognitive impairment (pMCI), and AD patients were constructed using the proposed individual metabolic network and compared with those from the group-based results. In addition, to illustrate the potential application, the network parameters, including the small-world network properties of network efficiency, clustering coefficient, gamma, and lambda in an individual network, and the network density score (NDS) of each generated network in the default-mode network (DMN) were calculated and compared among the AD disease groups.

## Materials and Methods

### Subjects

Data were obtained from the Alzheimer’s Disease Neuroimaging Initiative (ADNI) database^1^. The ADNI was launched in 2003 as a public–private partnership with the primary goal of testing whether serial magnetic resonance imaging (MRI), PET, other biological markers, and clinical and neuropsychological assessment can be combined to measure the progression of MCI and early AD. ADNI (ADNI ClinicalTrials.gov identifier: NCT00106899) is the result of the efforts of many coinvestigators from a broad range of academic institutions and private corporations, with subjects recruited from over 50 sites across the United States and Canada. Details of the ADNI-1 and ADNI-2 protocol, timelines, study procedures, and biomarkers can be found in the ADNI-1 and ADNI-2 procedures manual^2^. For up-to-date information, see www.adni-info.org.

To select age-matched subjects with significant clinical performance in different disease groups, PET data of 100 subjects consisting of 39 women and 61 men, 45 sMCI and 55 pMCI, and 100 AD subjects were included in this study. The demographic data was listed in Table 1. The definition of sMCI is for subjects with stable diagnosis of MCI at least for 36 months and pMCI if progression to AD within 12 months after baseline but no reversion to MCI or NC later.

**TABLE 1:** Summary of subject information.

Variable	Mean (±SD)			
	NC	sMCI	pMCI	AD
No. subjects	100	45	55	100
Gender (male/female)	61/39	32/13	34/21	58/42
Age (years)	75.6 (±4.3)	77.1 (±7)	75.4 (±6.4)	76 (±6.7)
Education (years)	15.1 (±3.2)	14.8 (±3.3)	15.8 (±2.9)	15.5 (±3.1)
MMSE	29.1 (±0.9)	27 (±4.6)	26 (±2.7)	21.9 (±4)
Global CDR	0.01 (±0.1)	0.47 (±0.1)	0.51 (±0.1)	0.93 (±0.5)

*Group demographics and clinical data upon 6-month follow-up.*

All image processing was performed using PMOD image analysis software (version 3.7; PMOD Technologies Ltd, Zurich, Switzerland). PET image was first spatially normalized into the Montreal Neurological Institute (MNI) space based on the FDG-template from the PMOD. Regional SUV ratio (SUVR; standard uptake value ratio) was calculated by using the whole cerebellum as the reference region (Hsiao et al., 2013). Finally, each subject’s regional SUVR for each AAL structure was extracted to construct the SUVR data matrix with a size of 1 × N, where “N” is the number of AAL structures including 90 regions (Jack et al., 2008). More details about the data processing information can be found in Huang et al. (2018). The details about the PET FDG imaging protocols can be obtained from the ADNI website^3^.

### Individual Metabolic Brain Network

The group-based metabolic network represents the average metabolic connectivity within a group (Huang et al., 2018), but loses the individual network information. In order to solve this problem, here we derived an individual metabolic network based on the ES difference of regional SUVR between a single subject and a NC group. The procedure is described below.

An ES is to measure the amount of association between two variables or differences between two groups in an experiment (Wilkinson, 1999; Nakagawa and Cuthill, 2007; Ellis, 2010; Kelley and Preacher, 2012). For example, Cohen’s *d* is defined as the difference between two group means divided by the standard deviation of the data as $d=\frac{{\bar{x}}_{1}-{\bar{x}}_{2}}{s}$, where *s* is the pooled standard deviation (Brand et al., 2011).

Here, to derive the individual network, we first modified a treatment effect measure from an independent-group pretest-posttest design (Becker, 1988; Morris and DeShon, 2002; Kadel and Kip, 2012) to calculate the regional difference between the single-subject SUVR deviation from the mean SUVR value of normal subjects in two regions. Let *x*_*i*_ and *x*_*j*_ be regional SUVRs for regions *i* and *j* from one subject, ${\bar{X}}_{NC,i}$ and ${\bar{X}}_{NC,j}$ be the mean regional SUVR of the normal group, and *s*_*i*_ and *s*_*j*_ be the corresponding standard deviations of the regional SUVRs. Then, the ES difference of the SUVR deviation between the *i*-th and *j*-th regions from corresponding regions in the normal group can be calculated as

(1) $$\text{ES}\left(i,j\right)=\frac{x_{i}-{\bar{X}}_{\mathrm{NC},i}}{s_{i}}-\frac{x_{j}-{\bar{X}}_{\mathrm{NC},j}}{s_{j}}.$$

For calculating the ES in the dependent paired group, Cohen (1988) suggested using the following pooled standard deviation, $s_{p}\left(i,j\right)=\sqrt{\frac{s_{i}^{2}+s_{j}^{2}}{2}}$. Using the pooled standard deviation and taking the absolute value of the difference, the corresponding ES difference between regions *i* and *j* in Eq.1 can be then modified as

(2) $$\text{ESD}\left(i,j\right)=\frac{\left|\left(x_{i}-{\bar{X}}_{\mathrm{NC},i}\right)-\left(x_{j}-{\bar{X}}_{\mathrm{NC},j}\right)\right|}{s_{p}\left(i,j\right)}.$$

**FIGURE 1:**
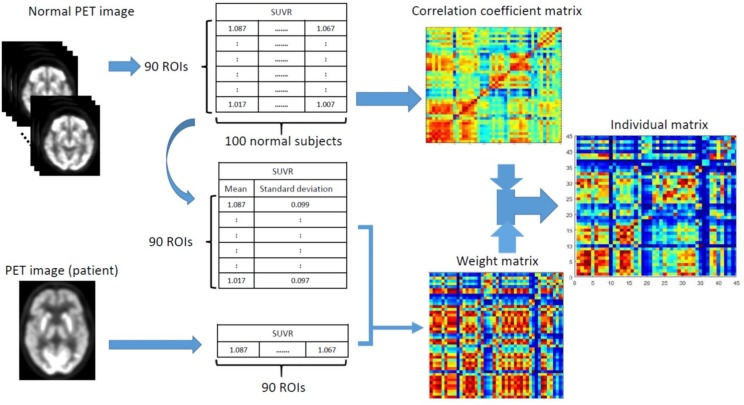
Schematic workflow for calculating an individual matrix of correlation coefficients by first calculating the individual weighting matrix (W) and correlation coefficient matrix (M_*NC*_) from the normal group, and then multiplying both W and M_*NC*_ to obtain the final individual correlation coefficient matrix.

The ESD (*i, j*) can be calculated for all pairs of ROI (*i, j*) to obtain a final ESD matrix (90 × 90). By viewing ESD (*i*, *j*) as *z* score (Kim, 2015) and applying simple Fisher transformation (Fisher, 1921) of $z=\frac{1}{2}\text{ln}\left(\frac{1+R}{1-R}\right)$, one can obtain

$$R=\frac{\exp\left(2z\right)-1}{\exp\left(2z\right)+1},$$

where *R* is the correlation coefficient. By applying the Fisher transformation, the correlation coefficient value *R* (*i*, *j*) between *i*-th and *j*-th regions can then be derived as

(3) $$R\left(i,j\right)=\frac{\exp\left(2\times \text{ESD}\left(i,j\right)\right)-1}{\exp\left(2\times \text{ESD}\left(i,j\right)\right)+1},$$

where 0 < *R* (*i, j*) < 1. A stronger difference of SUVR variation between two regions infers a higher ESD (*i, j*) value, which leads to a smaller single-subject regional correlation coefficient. However, the transformation formula (Eq. 3) will generate a higher value for *R* (*i, j*). Thus, to adjust this, we then applied the 1*-R* (*i, j*) as a weighting factor *W* (*i, j*) for the regional correlation coefficient between the single subject and the NC group as *W* (*i*, *j*) = 1-*R* (*i*, *j*). The final individual correlation matrix for a single subject with element *M* (*i, j*) between regions *i* and *j* was then calculated as

(4) $$M\left(i,j\right)=W\left(i,j\right)⊙M_{NC}\left(i,j\right)$$

where *M*_*NC*_ (*i, j*) is the group-based correlation coefficient between the *i*-th and *j*-th regions of the NC group, and *W* (*i, j*) = 1−*R* (*i, j*). The final individual correlation coefficient matrix (i.e., connectivity matrix) for a single subject can then be computed as *W*⊙*M*_*NC*,_ where *M*_*NC*_ is the group-based regional correlation coefficient matrix of the NC group, *W* is the weighting matrix, and ⊙ indicates an element-by-element multiplication. As shown in Figure 1, the final processing workflow of the individual metabolic network construction first calculates the individual weight matrix (*W*) and regional SUVR correlation coefficient matrix (*M*_*NC*_) from the normal group, and then multiplies both *W* and *M*_*NC*_ to obtain the final individual correlation coefficient matrix *M*. The processing steps were summarized as follows:

0. Perform spatial normalization and SUVR calculation for all FDG PET images.

1. Create correlation coefficient matrix of the NC group (*M*_*NC*_).

2. Calculate the mean (${\bar{X}}_{NC}$) and standard deviation (*s*) SUVR images in the NC group.

3. For given FDG image *x* from a subject, calculate the ES [ESD (*i*, *j*)] in Eq. 2.

4. Apply Fisher transformation to obtain correlation coefficient *R* in Eq. 3.

5. Calculate the weighting matrix *W = 1−R.*

6. Multiply W by *M*_*NC*_ in Eq. 4 to obtain the final individual correlation coefficient matrix *M*.

### Small-Worldness Analysis

To evaluate the network efficiency of our proposed individual brain network approach, small-world metrics were calculated (Latora and Marchiori, 2001; Achard and Bullmore, 2007; Wu et al., 2013). Here, we first measured the small-world parameters of the global efficiency, clustering coefficient, lambda, and gamma to evaluate the performance of individual brain networks for NC, pMCI, sMCI, and AD subjects.

The clustering coefficient is applied to measure the degree of connectivity among adjacent nodes. For any node, it is calculated as the number of edges that exist between its nearest neighbors. For a network with *N* nodes, the mean clustering coefficient *C* of the network is the average of the clustering coefficient over all nodes

(5) $$C=\frac{1}{N}\sum_{i}\frac{E_{i}}{D_{i}\left(D_{i}-1\right)/2},$$

where *D*_*i*_ is the number of all possible edges (neighbors) linking to the node *i* and *E*_*i*_ is the number of edges with direct links to node *i*.

Global efficiency (*Eg*) is a measure of the structure of a network (Wu et al., 2013) and can be computed as

(6) $$Eg=\frac{1}{N\left(N-1\right)}\sum_{i\neq j}\frac{1}{d_{ij}},$$

where *d*_*ij*_ is the shortest path length between nodes *i* and *j*.

A network could be defined as small-world network when gamma ≫ 1, lambda∼1, and sigma > 1. The two small-world characteristics for lambda and gamma are defined as λ = *L*/*Lr* and γ = *C*/*Cr* separately, where *Lr* and *Cr* indicates the clustering coefficient and the path length of the matched random networks. To achieve statistical significance, random networks were repeated for the network performance evaluation in this study 200 times (Chung et al., 2016; Chen et al., 2018; Yao et al., 2018). The measurement for lambda and gamma was conducted using the open toolkit GRETNA^4^ (Wang et al., 2015) and the Brain Connectivity Toolbox (BCT^5^) (Rubinov and Sporns, 2010).

### Network Density Scores

To investigate the performance of the proposed individual networks in different groups, the NDS of each generated network was calculated and compared. Here we only consider the subnetwork of the default mode network (DMN). For a given subject, the subnetwork was constructed from the subject’s binary matrix for a specified threshold (Qin et al., 2015).

The NDS was defined as the number of intra-network edges divided by all possible connections as

(7) $$C_{X}=\frac{1}{n_{X}\left(n_{X}-1\right)/2}\sum_{i,j=1:n_{X}}c_{i,j},$$

where *n*_*X*_ is the number of nodes within an intra-network *X* and *c*_*i,j*_ is the binary connectivity value between the *i*-th and *j*-th ROI after thresholding. For each pair of subnetworks between *X* and *Y*, the inter-network connectivity score was defined as

(8) $$C_{X,Y}=\frac{1}{n_{X}n_{Y}}\sum_{i\in X,j\in Y}c_{i,j}$$

### Statistical Analysis

ANOVA with *post hoc* Student’s *t*-test was applied to compare the network performance among the four groups, where the *post hoc* test (multiple comparisons) was used to identify the significant pair(s) and a significant *p*-value of the *post hoc* test indicates at least one pair with statistically significant mean difference. All the statistical analyses were performed by using the SPSS package (SPSS Statistics for Windows, version 17.0, 2008), and significance testing of *p* < 0.05 and *p* < 0.01 was applied.

## Results

### Inter-Hemispheric Correlation Coefficients Matrices

To build a connectivity network, a connectivity matrix is usually converted to a binary matrix by a threshold (Huang et al., 2018), where the links above the threshold are represented by one (presence of edge) and those below it are represented by zero (absence of edge). Here, to reduce the complexity of visualizing the connectivity network, the lowest threshold value for the connectivity map in the NC containing 90 nodes was selected for all groups in this study, and this led to the threshold of 0.4354, which was applied for all subsequent processing. Note that all the group-level analysis result was performed based on our previous work (Huang et al., 2018).

**FIGURE 2:**
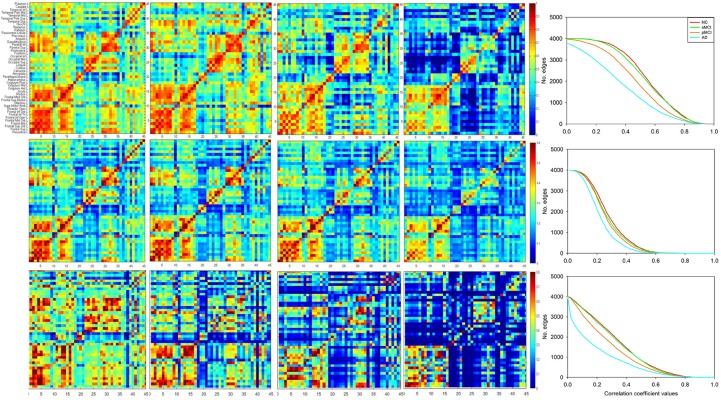
Matrices of correlation coefficients between the right and left hemisphere (ordinate) brain regions. The inter-hemispheric connectivity is illustrated from the matrices of correlation coefficients between the right hemisphere (abscissa) and left hemisphere (ordinate) brain regions for NC, sMCI, pMCI, and AD, as obtained via the conventional group-based method (first row), average individual connectivity matrices (second row), and anecdotal single-subject connectivity matrices (third row) with the proposed method. The last column illustrates the plots for number of edges (connectivity) vs. varying thresholds at each disease group for the corresponding matrix of correlation coefficients.

To compare with the conventional group-based connectivity matrix, Figure 2 displays the group-based conventional inter-hemispheric connectivity matrices of the correlation coefficients from the NC, sMCI, pMCI, and AD groups (first row) (Huang et al., 2018), the average individual inter-hemispheric connectivity matrices obtained by the proposed method from the NC, sMCI, pMCI, and AD groups (second row), and the anecdotal single-subject connectivity matrices from single NC, sMCI, pMCI, and AD subjects (third row). From the group-based connectivity matrices in the first row, the main connection differences among the groups are in the temporal, parietal, and occipital lobes. Similar results can be observed in the average single-subject connectivity matrices in the second row, and also the anecdotal single-subject matrices in the third row for NC, sMCI, pMCI, and AD, respectively. From all the matrices, the connectivity matrix for NC displays more connections with statistical significance as compared to AD, where AD shows a more obvious decrease in connections between the frontal lobe and other regions. The connectivity patterns are similar for both sMCI and NC, while the pattern of connectivity reduction in pMCI falls between those of sMCI and AD.

Moreover, the last column in each row illustrates the plots for number of edges (connectivity) vs. varying thresholds at each disease group for the corresponding matrix of correlation coefficients from each row. Under the same threshold, NC displays more connections as compared to other groups and is followed by sMCI and pMCI, while AD shows significant reduction of connectivity for both group-level and individual networks.

### Inter-Hemispheric Connectivity Network

**FIGURE 3:**
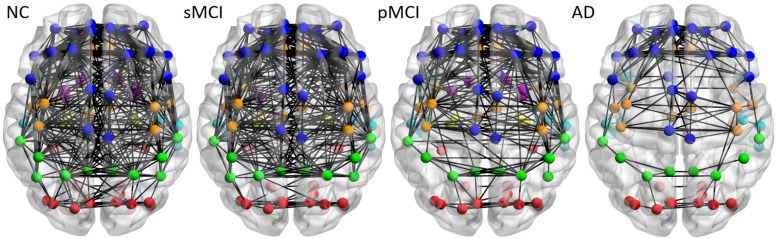
The axial view of mean individual inter-hemispheric brain connectivity graphs of NC, sMCI, pMCI, and AD constructed using the proposed individual network approach. The inter-hemispheric connectivity graphs were visualized for four groups and obtained by thresholding the average correlation coefficient matrix from individuals of each group using the threshold of 0.4354, since this value offers the highest correlation coefficient with all connections (90 nodes) in NC. The inter-hemispheric connections are indicated by black lines and nodes are represented by different colors (deep blue for frontal; light blue for temporal; green for parietal; red for occipital; pink for thalamus, pallidum, caudate, putamen, amygdala; yellow for hippocampus; deep yellow for other regions).

Figure 3 illustrates the axial view of the average individual inter-hemispheric connectivity network built from a binary matrix as measured from the same correlation coefficient threshold for all four groups, and obtained from the connectivity matrices in the second row of Figure 2. The connections are displayed by black lines and nodes are shown by the color dots (deep blue for frontal; light blue for temporal; green for parietal; red for occipital; pink for thalamus, pallidum, caudate, putamen, amygdala; yellow for hippocampus; deep yellow for other regions). The sMCI subjects show similar patterns of inter-hemispheric connectivity to those in NC, but with slightly reduced inter-lobe connections. However, as compared to pMCI, the network connectivity for sMCI was significantly higher in the frontal and parietal-frontal lobes. The AD group showed significantly reduced connections in all regions among the four groups. As shown in Supplementary Table 1, the resulting number of edges in the inter-hemispheric connectivity network for each group is 213, 207, 142, and 61 for NC, sMCI, pMCI, and AD, respectively. One can observe a significant reduction of inter-hemispheric connections in pMCI and AD.

### Intra-Hemispheric Connectivity Network

**FIGURE 4:**
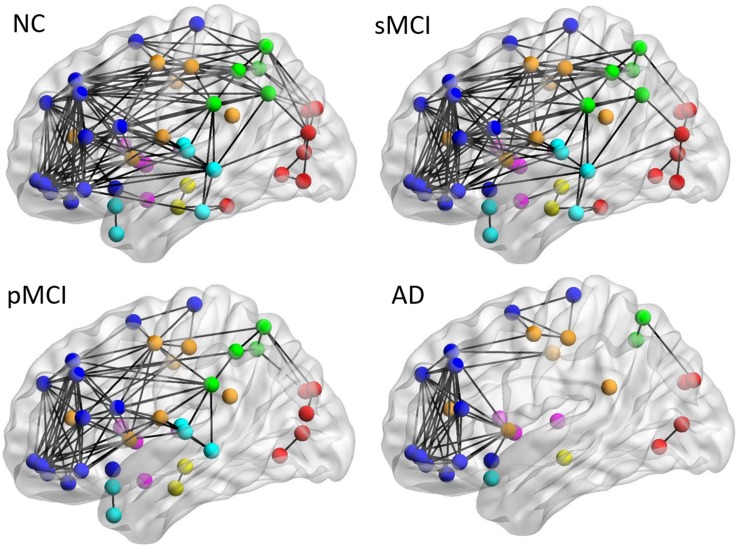
The lateral view of mean individual intra-hemispheric connectivity graphs of NC, sMCI, pMCI, and AD. The intra-hemispheric connectivity graphs were visualized for four groups and obtained by thresholding the correlation coefficient matrix using the threshold of 0.4354. The intra-hemispheric connections are indicated by black lines and nodes are represented by different colors (deep blue for frontal; light blue for temporal; green for parietal; red for occipital; pink for thalamus, pallidum, caudate, putamen, amygdala; yellow for hippocampus; deep yellow for other regions).

Figure 4 shows the lateral view of the average individual intra-hemispheric connectivity network for each group, as built from the connectivity matrices in the second row of Figure 2. Significant intra-hemispheric connectivity reductions in the frontal, temporal, parietal, and occipital regions were observed for both pMCI and AD, and in particular between the temporal and parietal regions, frontal and temporal regions, and also temporal and occipital regions. The intra-hemispheric connectivity pattern was similar for both sMCI and NC, except for the slightly reduced connection between parietal and occipital found in sMCI. As shown in the Supplementary Table 1, the resulting number of edges in the intra-hemispheric connectivity network using the individual method is 161, 143, 103, and 77 for NC, sMCI, pMCI, and AD, respectively.

### Metabolic Connectivity Network Differences

**FIGURE 5:**
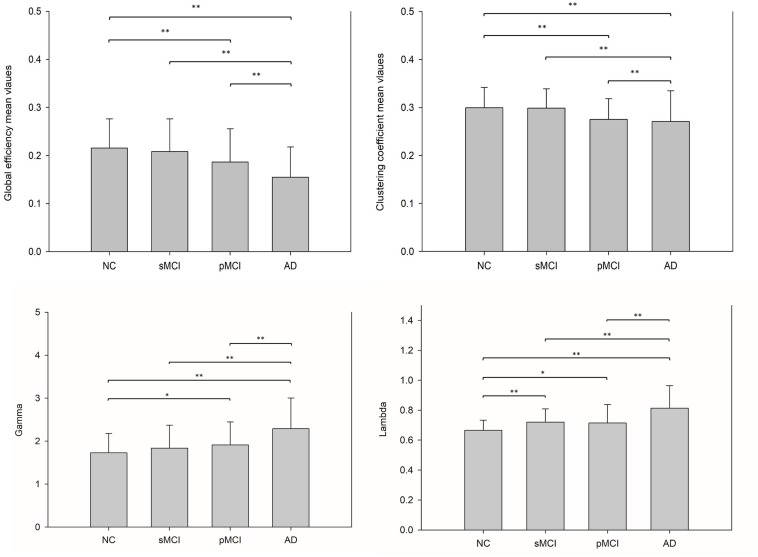
This illustrates the bar plots of the global efficiency, clustering coefficient, gamma and lambda calculated from each individual metabolic network for NC, sMCI, pMCI, and AD. A reduction trend was observed for both global efficiency and clustering coefficient starting from NC, sMCI, pMCI, and AD, while a reverse reduction trend for gamma and lambda values. *p < 0.05, **p < 0.01.

The small-worldness characteristic parameters were calculated from the proposed individual connectivity networks for each subject, and scatter plots were plotted for each group as shown in Figure 5. Reduction trends in global efficiency and clustering coefficients were observed for NC, sMCI, pMCI, and AD. Global efficiency and clustering coefficients were statistically different between NC and AD, and also between NC and pMCI (*p* < 0.01, uncorrected). However, neither global efficiency or clustering coefficients were statistically different when comparing sMCI to NC.

### Network Density Score Differences

**FIGURE 6:**
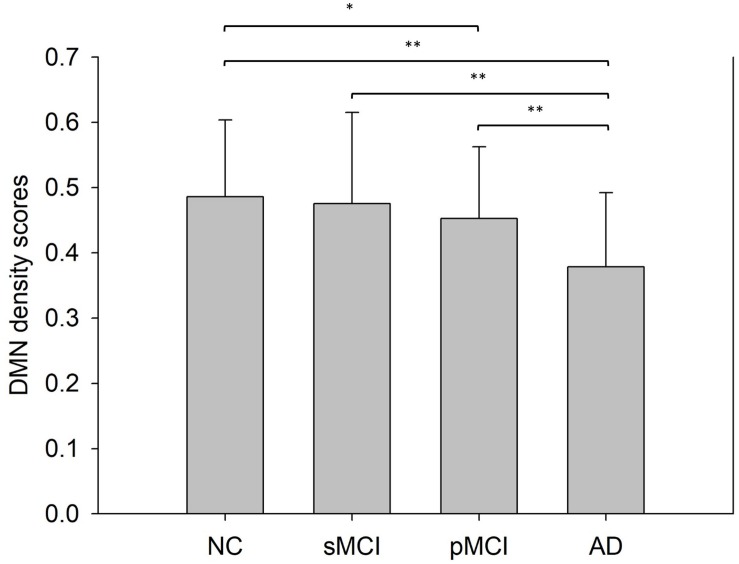
This shows the mean network density scores (NDS) of the default mode network intra-subnetwork from a Student’s *t*-test with significance indicated by *p < 0.05 and **p < 0.01.

Figure 6 shows the bar plots of NDS score in the DMN subnetwork calculated from the proposed individual network for the four groups. The result shows that the mean NDS decreased in the DMN subnetwork from NC to AD. Also, no significant NDS differences were observed between NC and sMCI, or between pMCI and AD.

## Discussion

In this study, we developed a novel approach for building an individual metabolic network using a single-subject FDG PET image and NC database. The results show the feasibility of the proposed individual brain network for generating network properties and differentiating different disease groups of dementia, including NC, sMCI, pMCI, and AD. The proposed individual metabolic network also produced similar network features to those derived from the group-based brain metabolic network (Huang et al., 2018). In particular, the key patterns of occipital–parietal inter-regional connectivities detected in the group-based network study for differentiating different disease groups in AD were also found in the individual network.

Our proposed method for individual network construction is based on the multiplication of a weighting factor and the group-based connectivity matrix calculated from a group of normal subjects. Under normal conditions, the structure of the brain network is a stable and highly relevant link. When the metabolism of the brain changes in a subject due to disease, the network link will change accordingly. Each region may exhibit different levels of variation in metabolism, and that leads to deviation from the normal value in the control group. Thus, the weighting factor for adjusting the average normal group link was based on the ES difference of the regional SUVR deviation of a single subject from the NC group. This was first modified from a method for evaluating the overall treatment effect of different treatments using an independent-group pretest-posttest design (Becker, 1988), where different groups receive different treatments (e.g., experimental and control groups), and outcomes are measured both before and after the treatment. The ES within each treatment condition is calculated, and then the ES difference between the control group and the experimental group, as in our equation, is applied to compute the overall treatment ES (Becker, 1988; Morris and DeShon, 2002). In our approach, after a simple Fisher transformation, the ES difference was converted into a correlation value, and then a weighting factor. The idea is that the higher the inter-regional SUVR effect-size difference between the single-subject and the normal group, the lower the similarity of the regional correlation coefficient in a given subject to that of the normal group.

In our previous group-based network study (Huang et al., 2018), we found two key patterns in identifying whether MCI presents a high risk of progression to AD or not, namely parieto-occipital connectivity for sMCI and pMCI, and temporal-occipital connectivity for NC and MCI. The pattern of parieto-occipital connectivity was also observed in the individual metabolic network. For example, the connectivity density between parietal and occipital lobes was slightly higher in NC and pMCI but not in sMCI (Figure 4); a similar result was also shown in the group network (Huang et al., 2018). However, there is only a slight increase but no significant connectivity between the frontal and parietal connection in sMCI as compared to NC. Interestingly, the possible compensatory effect of increased left frontal connectivity in AD from the group-based network was also shown in the individual one (Figure 4 and Supplementary Table 1). For the average number of edges for inter- and intra-hemispheric networks for individual metabolic networks, as shown in Supplementary Table 1, there were much fewer inter- and intra-hemispheric connections in the AD group as compared to other groups. Similar results can be viewed in the connectivity graphs shown in Figures 3 and 4. More study on individual network connectivity pattern for each single subject is necessary and is the future goal.

Connection efficiency of network structure, clustering efficiency of average network clustering, lambda, and gamma can be used to characterize the patterns of network connectivity (Rubinov and Sporns, 2010). Our results showed that the global efficiency and clustering coefficient using the individual metabolic network method display reduction trends for NC, sMCI, pMCI to AD, and a reverse reduction trend for gamma and lambda. This falls in line with previous studies showing network efficiency reductions in MCI as compared to NC (Achard and Bullmore, 2007; Bullmore and Sporns, 2012) and a decrease in the clustering coefficient in AD when (Yong et al., 2009) using fMRI analysis.

The activity in the default mode network (DMN), which represents the resting state of the brain, has been proved to be a sensitive and specific biomarker for AD using fMRI (Greicius et al., 2004). Koch et al. (2012) observed declining trends in DMN connectivity in NC, MCI, and AD. The DMN derived from our individual network also displayed the same decreasing trend among the NC, sMCI, pMCI, and AD disease groups (Figure 6).

Although the correlation of node connections based on mutual information (MI) can show a nonlinear relationship (Wang et al., 2016; Jiang et al., 2017), there are also reports that FDG has strong connectivity in some areas (Huang et al., 2018). Through MI calculations, these connections are less likely to be highlighted, and our correlation matrix makes it is clear that there are areas of higher correlation.

A metabolic network may provide probable imaging biomarkers of neurodegenerative disease to identify those at higher risk in developing neurodegenerative disorders (Wu et al., 2013). For conventional network analysis based on correlation coefficient matrix, a threshold is needed. However, an optimal choice of threshold value is challenging and it is possible to miss important diagnosis information after thresholding. Since our individual method provides more convenient and powerful diagnosis power for clinical application, analysis based on threshold-free approach as in Kuang et al. (2019) might help to preserve more information. Future work should include the investigation of the threshold-free analysis.

For our current study, age-matched NC subjects and disease groups were used for constructing the NC group data and for evaluation of the individual network. In theory, a connectivity pattern is age-dependent; therefore, future work should study the age effect of group NC connectivity matrices in the individual networks of different age groups. One of the limitations in this work is the small sample size, and future work should include more subjects to investigate individual metabolic network variability and derive the connectivity spectrums in different groups for clinical diagnosis purposes. Another limitation is the segmentation or parcellation error introduced by the spatial normalization method based on FDG-template as compared to MRI-based approach in this study. Finally, our proposed individual network approach can be applied to other PET tracers for molecular connectivity (Sala and Perani, 2019).

## Conclusion

We proposed a novel method for constructing an individual metabolic network using a single-subject FDG PET image and a NC FDG PET database. The result has shown the effectiveness and feasibility of the proposed individual metabolic network in differentiating different AD disease groups. Future studies should investigate inter-individual variability and the correlations of individual network features to disease severities and clinical performance.

## Data Availability Statement

PET images were downloaded online from ADNI (https://ida.loni.usc.edu) and further processed locally (see Image Analysis above). Processed ADNI data are not publicly available for download but are available from the corresponding author.

## Ethics Statement

All methods were performed in accordance with the relevant ethical guidelines and regulations as stated in the first section of Materials and Methods. Since the data were obtained from the ADNI database, no written inform consent is required according to local legislation and national guidelines.

## Author Contributions

S-YH, J-LH, and I-TH designed the study. S-YH, K-JL, and I-TH analyzed the data. S-YH and I-TH wrote the manuscript. All authors revised and approved for publication.

## Conflict of Interest

The authors declare that the research was conducted in the absence of any commercial or financial relationships that could be construed as a potential conflict of interest.

## Footnotes

1 adni.loni.usc.edu

2 http://www.adni-info.org/

3 www.loni.ucla.edu/ADNI

4 https://www.nitrc.org/projects/gretna/

5 http://www.nitrc.org/projects/bct/
