# Supplementary material for: A Novel Individual Metabolic Brain Network for 18F-FDG PET Imaging
Source: Front Neurosci. 2020 May 12;14:344. doi: 10.3389/fnins.2020.00344 (PMC7235322; doi:10.3389/fnins.2020.00344)
Supplement: Supplementary file 1 [file Table_1.pdf]

Supplementary Material

1 Supplementary Tables

Supplementary Table 1. Average number of edges for inter and intra-hemispheric connectivity calculated from individual metabolic network at each group.

No. edges	NC	sMCI	pMCI	AD
LL	161	143	103	77
RR	139	114	103	51
LR	213	207	142	61
Total	513	464	348	189
No. nodes	90	90	87	80

L-L: Left intra-hemisphere, **R-R:** Right intra-hemisphere, and **L-R: Left and right i**nter-hemisphere
